# Supplementary material for: Intergenerational effects of parental educational attainment on parenting and childhood educational outcomes: Evidence from MoBa using within-family Mendelian randomization
Source: medRxiv. 2023 Mar 22:2023.02.22.23285699. Originally published 2023 Feb 22. Preprint. [Version 2] doi: 10.1101/2023.02.22.23285699 (PMC9980223; doi:10.1101/2023.02.22.23285699)
Supplement: Supplementary file 1 [file media-2.docx]

**Intergenerational effects of parental educational attainment on parenting and childhood educational outcomes: Evidence from MoBa using within-family Mendelian randomization**

Alexandra Havdahl^1,2,3,^^, Amanda M Hughes^1,4,^^, Eleanor Sanderson^1,4^, Helga Ask^2,5^, Rosa Cheesman^5^, Ted Reichborn-Kjennerud^6^, Ole A. Andreassen^7^, Elizabeth C Corfield^2,3^, Laurie Hannigan^1,2,3^, Per Magnus^6^, Pål Njølstad^8^, Camilla Stoltenberg^6^, Fartein Ask Torvik^5,9^, Ragnhild Brandlistuen^6^, George Davey Smith^1,4^, Eivind Ystrom^1,2,3,5^, and Neil M Davies^1,10,11,12,*^

^1^ Medical Research Council Integrative Epidemiology Unit at the University of Bristol, BS8 2BN, United Kingdom.

^2^ Department of Mental Disorders, Norwegian Institute of Public Health, Oslo, Norway.

^3 ^Nic Waals Institute, Lovisenberg Diaconal Hospital, Oslo, Norway.

^4 ^Population Health Sciences, Bristol Medical School, University of Bristol, Barley House, Oakfield Grove, Bristol, BS8 2BN, United Kingdom.

^5^ Department of Psychology, PROMENTA Research Center, University of Oslo, Oslo, Norway

^6^ Department of Child Health and Development, Norwegian Institute of Public Health, Oslo, Norway

^7 ^NORMENT Centre, Division of Mental Health and Addiction, Oslo University Hospital &

Institute of Clinical Medicine, University of Oslo, Oslo, Norway

^8^ University of Bergen, Bergen, Norway

^9^ Centre for Fertility and Health, Norwegian Institute of Public Health, Oslo, Norway

^10 ^K.G. Jebsen Center for Genetic Epidemiology, Department of Public Health and Nursing, Norwegian University of Science and Technology, Norway.

^11^ Division of Psychiatry, University College London, Maple House, 149 Tottenham Court Rd, London W1T 7NF

^12^ Department of Statistical Sciences, University College London, London WC1E 6BT, UK

**Supplementary materials**

#### **Supplementary Figure 1: STROBE Flow chart of inclusion and exclusion from the study**

#### **Supplementary Figure 2: Sex stratified FEMALES Effect of parents’ educational attainment on children’s nationally standardised test scores, estimated using multivariable-adjusted regression (OLS) and within-family Mendelian randomization (WF), estimated on the full sample using multiple imputation (N=20,886). Mean difference in standard deviations and 95% confidence intervals in test scores per year of parental education reported.**

#### **Supplementary Figure 2: Sex stratified MALES Effect of parents’ educational attainment on children’s nationally standardised test scores, estimated using multivariable-adjusted regression (OLS) and within-family Mendelian randomization (WF), estimated on the full sample using multiple imputation (N=20,021). Mean difference in standard deviations and 95% confidence intervals in test scores per year of parental education reported.**

#### **Supplementary Figure 3:  Sex-stratified FEMALES Effect of parents’ educational attainment on questionnaire measures of reading and communication skills and parental nurturing at age 5, estimated using multivariable-adjusted regression (OLS) and within-family Mendelian randomization (WF), estimated on the full sample using multiple imputation (N=20,886).**

#### **Supplementary Figure 4:  Sex-stratified MALES Effect of parents’ educational attainment on questionnaire measures of reading and communication skills and parental nurturing at age 5, estimated using multivariable-adjusted regression (OLS) and within-family Mendelian randomization (WF), estimated on the full sample using multiple imputation (N=20,021).**

#### **Supplementary Figure 6:  Sex stratified FEMALES Effect of parents’ educational attainment on questionnaire measures of early educational attainment and parental nurturing at age 8, estimated using multivariable-adjusted regression (OLS) and within-family Mendelian randomization (WF), estimated on the full sample using multiple imputation (N=20,886).**

#### **Supplementary Figure 7:  Sex stratified MALES Effect of parents’ educational attainment on questionnaire measures of early educational attainment and parental nurturing at age 8, estimated using multivariable-adjusted regression (OLS) and within-family Mendelian randomization (WF), estimated on the full sample using multiple imputation (N=20,021).**

#### **Supplementary Figure 8: Simulation comparing ordinary least squares (OLS), standard Mendelian randomization and within-family Mendelian randomization estimates of the effect of mothers on their children. The true effect size of the dynastic effect is one, indicated by the red line. Within-family Mendelian randomization estimators can provide an unbiased estimate of the effects of parents on their offspring, even in the presence of assortative mating. At very high levels of assortment, the precision of these estimators falls. OLS and standard Mendelian randomization are biased.**
